# Comparison of influenza vaccine effectiveness in preventing outpatient and inpatient influenza cases in older adults, northern Spain, 2010/11 to 2015/16

**DOI:** 10.2807/1560-7917.ES.2018.23.2.16-00780

**Published:** 2018-01-11

**Authors:** Jesús Castilla, Iván Martínez-Baz, Ana Navascués, Itziar Casado, Aitziber Aguinaga, Jorge Díaz-González, Josu Delfrade, Marcela Guevara, Carmen Ezpeleta, Primary Health Care Sentinel Network of Navarre, Network for Influenza Surveillance in Hospitals of Navarre

**Affiliations:** 1 Instituto de Salud Pública de Navarra, IdiSNA - Navarre Institute for Health Research, Pamplona, Spain; 2 CIBER Epidemiología y Salud Pública (CIBERESP), Madrid, Spain; 3 Complejo Hospitalario de Navarra, IdiSNA - Navarre Institute for Health Research, Pamplona, Spain; 4 The members of the networks are listed at the end of the article

**Keywords:** Influenza virus, influenza vaccine, vaccine effectiveness, hospitalised influenza, case-control study

## Abstract

We compared trivalent inactivated influenza vaccine effectiveness (VE) in preventing outpatient and inpatient influenza cases in Navarre, Spain. **Methods:** During seasons 2010/11 to 2015/16, community-dwelling patients with influenza-like illness aged 50 years or older were tested for influenza when attended by sentinel general practitioners or admitted to hospitals. The test–negative design was used to estimate and compare the VE by healthcare setting. **Results:** We compared 1,242 laboratory-confirmed influenza cases (557 outpatient and 685 inpatient cases) and 1,641 test-negative controls. Influenza VE was 34% (95% confidence interval (CI): 6 to 54) in outpatients and 32% (95% CI: 15 to 45) in inpatients. VE in outpatients and inpatients was, respectively, 41% (95% CI: –1 to 65) and 36% (95% CI: 12 to 53) against A(H1N1)pdm09, 5% (95% CI: –58 to 43) and 22% (95% CI: –9 to 44) against A(H3N2), and 49% (95% CI, 6 to 73) and 37% (95% CI: 2 to 59) against influenza B. Trivalent inactivated influenza vaccine was not associated with a different probability of hospitalisation among influenza cases, apart from a 54% (95% CI: 10 to 76) reduction in hospitalisation of influenza A(H3N2) cases. **Conclusions:** On average, influenza VE was moderate and similar in preventing outpatient and inpatient influenza cases over six influenza seasons in patients above 50 years of age. In some instances of low VE, vaccination may still reduce the risk of hospitalisation in older adults with vaccine failure.

## Introduction

Annual influenza epidemics are associated with large numbers of medical consultations and hospitalisations. Most influenza patients are seen in general practice, but sometimes influenza evolves to serious forms or worsens underlying chronic conditions and requires hospitalisation. Certain risk groups and older adults with influenza are at increased risk of hospitalisation [1-3].

In most seasons, influenza vaccine is moderately effective (around 50%) in preventing influenza cases [4-9]; however, there is little information on whether it is equally effective in preventing outpatient and inpatient (hospitalised) cases [10]. Vaccine effectiveness (VE) estimates obtained from the general practice and hospital settings in the same area and season may be expected to be similar since they evaluate the same types of influenza vaccine against the same circulating virus. However, inpatient cases tend to be older and to present more underlying chronic conditions, so they are more likely to be affected by immunodepression and immunosenescence, which could reduce the influenza vaccine effect [11]. In studies, most of these factors can be controlled for in multivariate analyses. However, a higher VE could be observed in hospitalised patients if the influenza vaccine mitigates influenza illness severity, reducing the risk of hospital admission in people in whom it did not prevent influenza infection [8].

The test-negative case–control design that compares laboratory-confirmed influenza cases and test-negative controls [12], has been widely used to evaluate influenza VE in preventing laboratory-confirmed influenza. This design was employed to evaluate outpatient [13-16] and inpatient cases [17,18]. However, very few studies obtained influenza VE estimates in the general practice and hospital settings for the same influenza season and population [19-21], and none of them provided conclusive comparisons of both estimates [10]. As influenza VE may differ from season to season, pooled analysis of several seasons is used to obtain average estimates [15-22].

Our test-negative case–control study nested in a population-based cohort aims to estimate trivalent inactivated influenza VE in preventing outpatient and inpatient cases with laboratory-confirmed influenza among community dwelling adults aged 50 years or older throughout six influenza seasons, and to identify possible differences in VE between the general practice and hospital settings.

## Methods

### Study population and setting

This study was performed in the region of Navarre (642,000 inhabitants), Spain, during the influenza seasons 2010/11 to 2015/16. Six seasons were included to assure a larger size of the study population and periods with circulation of the influenza viruses A(H1N1)pdm09, A(H3N2) and B. The Navarre Health Service provides primary and hospital healthcare, free of charge, to over 95% of the population. Since 2009, influenza VE has been evaluated annually, using a test negative case–control design nested in a population-based cohort, including patients from sentinel general practitioners and hospitals [21,23-25]. The Navarre Ethical Committee for Medical Research approved the study protocol.

Each influenza season, the non-adjuvanted inactivated trivalent vaccine was recommended and offered free of charge to people aged 60 years or older, and to patients with major chronic conditions (heart disease, lung disease, renal disease, cancer, diabetes mellitus, liver cirrhosis, dementia, cerebral stroke, immunocompromised, and body mass index of 40 kg/m^2^ or greater) [26]. The vaccination programme distributed a single brand of trivalent inactivated vaccine in each influenza season. People not covered by the recommendation could also be vaccinated if they purchased the vaccine in pharmacies.

Influenza surveillance was based on automatic reporting from the electronic medical records of all cases of influenza-like illness (ILI) diagnosed in primary healthcare centres and hospitals. ILI was considered to be the sudden onset of any general symptom (fever or feverishness, malaise, headache or myalgia) in addition to any respiratory symptom (cough, sore throat or shortness of breath) [27]. A sentinel network composed of a representative sample of 76 to 80 primary healthcare physicians was asked to take double swabs, nasopharyngeal and pharyngeal, from all patients diagnosed with ILI whose symptoms had begun less than 5 days previously. The protocol for influenza management in hospitals establishes early detection and nasopharyngeal and pharyngeal swabbing of all hospitalised patients with ILI. Swabs were processed by reverse transcription PCR (RT-PCR) assay in the microbiology departments of the Navarre Hospital Complex and the Navarre University Clinic.

The present study included ILI cases aged 50 years or older attended in primary healthcare (outpatients) or hospitalised (inpatients) who were swabbed during the study period. Healthcare workers, persons living in nursing homes, patients hospitalised less than 24 hours, and patients who were hospitalised before ILI symptom onset were excluded. For each influenza season, the study period was defined as November to May of the following year.

Influenza vaccine status was obtained from the regional vaccination register. Subjects were considered to be protected 14 days after vaccine administration.

The information on sex, age, major chronic conditions (heart disease, respiratory disease, renal disease, cancer, diabetes mellitus, liver cirrhosis, dementia, cerebral stroke, immunocompromised, rheumatic disease, and body mass index ≥ 40 kg/m^2^), functional dependence (Barthel index score < 40), previous hospitalisation, and month of sample collection was obtained from the electronic clinical records.

### Study design and statistical analysis

A double test-negative case–control design in outpatients and inpatients, both nested in the same population-based cohort, was used to compare the vaccination status of ILI patients with laboratory-confirmed influenza and test-negative controls.

Percentages were compared by chi-squared test. Logistic regression models were used to obtain crude and adjusted odds ratios (OR) for influenza vaccination with their 95% confidence interval (CI). In addition to healthcare setting (primary or hospital), the adjusted models included sex, age group (50–59, 60–69, 70–79, and ≥ 80 years), major chronic conditions (0, ≥ 1), functional dependence, hospitalisation in the previous 12 months, and influenza season and month of sample collection. VE was estimated as a percentage: (1 – OR) x 100.

Cases were compared with controls recruited in the same healthcare setting to obtain VE estimates for all patients and separately for outpatient and inpatient cases. Separate analyses were also carried out by age group (50–64 years and ≥ 65 years), influenza (sub)type, presence of major chronic conditions, and influenza season. Although stratum-specific VE estimates were obtained from stratified models, we also tested the statistical significance of the interaction term among these variables and vaccine status.

The VE estimates obtained from outpatients and inpatients may not be comparable since these two groups usually differ in many characteristics. Therefore, in an adjusted analysis we compared the odds of vaccination of inpatient cases vs outpatient cases to estimate the VE in reducing the risk of hospital admission in people in whom the vaccine failed to prevent influenza infection.

## Results

### Description of cases and controls

During the six influenza seasons studied, 930 ILI patients attended primary healthcare and 1,953 ILI hospitalised patients were swabbed, and 557 (60%) and 685 (35%) were confirmed for influenza virus, respectively. Three virus (sub)types were widely represented: influenza A(H1N1)pdm09 (n=500 cases; 40%) , influenza A(H3N2) (n=474; 38%) and influenza B (n=264; 21%). Nine patients were coinfected with two viruses. The proportion of influenza A(H1N1)pdm09 virus infections was higher among inpatient than outpatient cases (44% (303/685) vs 34% (194/557); p = 0.001), while the proportion of influenza A(H3N2) virus infections (36% (245/685) vs 41% (226/557); p = 0.070) and influenza B (19% (125/685) vs 24% (133/557); p = 0.030) was lower among inpatients.

Among primary healthcare patients, cases and controls did not differ statistically in the distribution of the analysed covariables. However, in hospitalised patients, the percentage of those aged 80 years or older and of those hospitalised in the previous 12 months was higher among controls than in cases (Table 1).

**Table 1:** Characteristics of laboratory-confirmed influenza cases and test-negative controls in northern Spain, 2010/11 to 2015/16

	All laboratory-confirmed influenza cases		All test-negative controls			Outpatient cases		Outpatient controls			Inpatient cases		Inpatient controls		
	n	%	n	(%)	p value^a^	n	%	n	%	p value^a^	n	%	n	%	p value^a^
**Age groups (years)**															
50–59	412	33	354	22	<0.001	291	52	182	49	0.148	121	18	172	14	0.023
60–69	318	26	353	22		173	31	106	28		145	21	247	20	
70–79	268	22	419	26		81	15	72	19		187	27	347	27	
≥ 80	244	20	515	32		12	2	13	4		232	34	502	40	
**Sex**															
Male	622	50	866	53	0.152	258	46	175	47	0.838	364	53	691	54	0.589
Female	620	50	775	47		299	54	198	53		321	47	577	46	
**Major chronic conditions**															
No	459	37	414	25	<0.001	336	60	203	54	0.079	123	18	211	17	0.453
Yes	783	63	1,227	75		221	40	170	46		562	82	1,057	83	
**Hospitalisation in the previous 12 months**															
No	1,008	81	1,131	69	<0.001	513	92	348	93	0.440	495	72	783	62	<0.001
Yes	234	19	510	31		44	8	25	7		190	28	485	38	
**Functional dependence**															
No	1,210	97	1,589	97	0.349	557	100	373	100	NA	653	95	1,216	96	0.549
Yes	32	3	52	3		0	0	0	0		32	5	52	4	
**Influenza vaccine status**															
Unvaccinated	764	61	766	47	<0.001	412	74	252	68	0.033	352	51	514	40	<0.001
Vaccinated	478	39	875	53		145	26	121	32		333	49	754	60	
**Influenza season**															
2010/11	100	8	237	14	<0.001	50	9	55	15	0.061	50	7	182	14	<0.001
2011/12	129	10	116	7		112	20	62	17		17	3	54	4	
2012/13	90	7	131	8		66	12	52	14		24	4	79	6	
2013/14	279	23	314	19		93	17	53	14		186	27	261	21	
2014/15	279	23	313	19		114	21	67	18		165	24	246	19	
2015/16	365	29	530	32		122	22	84	23		243	36	446	35	
**Total**	1,242	100	1,641	100		557	100	373	100		685	100	1,268	100	

NA: not applicable.

^a^ Chi-squared test.

Compared with outpatient cases, hospitalised cases were more frequently aged 70 years or older (61% (418/685) vs 17% (93/557); p < 0.0001), had major chronic conditions (82% (562/685) vs 40% (221/557); p < 0.0001), and had a history of hospitalisation in the previous 12 months (28% (190/685) vs 8% (44/557), p < 0.0001).

None of the patients included in the study had received antiviral treatment before swabbing or hospitalisation.

### Influenza vaccine effectiveness by influenza season

The influenza VE was moderate to high (37% to 71%) in preventing laboratory-confirmed influenza in the seasons 2010/11 and 2015/16, both characterised by a predominance of influenza A(H1N1)pdm09 virus, as well as in the 2012/13 season dominated by influenza B. However, the VE was low to moderate (2% to 53%) in the 2011/12, 2013/14 and 2014/15 seasons, when the influenza A(H3N2) virus circulated widely. In the 2013/14 and 2014/15 seasons the estimates of influenza VE were null in preventing outpatient cases and were somewhat higher in inpatient cases. In three influenza seasons (2010/11, 2011/12 and 2015/16) the point estimate of VE was higher in outpatients, and in other three seasons (2012/13, 2013/14 and 2014/15), it was higher among inpatients (Table 2).

**Table 2:** Influenza vaccine effectiveness in preventing laboratory-confirmed cases by season and healthcare setting in northern Spain, 2010/11 to 2015/16

	2010/11	2011/12	2012/13	2013/14	2014/15	2015/16
**All patients**						
Cases, n (% vaccinated)	100 (27)	129 (29)	90 (19)	279 (45)	279 (47)	365 (38)
Influenza A(H1N1)pdm09	95	0	22	95	6	279
Influenza A(H3N2)	0	117	6	177	137	34
Influenza B	5	12	62	0	133	46
Controls, n (% vaccinated)	237 (52)	116 (41)	131 (47)	314 (56)	313 (53)	530 (57)
Crude VE, % (95% CI)	66 (43 to 79)	43 (3 to 66)	73 (50 to 86)	35 (9 to 53)	21 (–10 to 42)	53 (39 to 64)
Adjusted VE, % (95% CI) ^a^	53 (14 to 74)	53 (–9 to 79)	71 (35 to 87)	21 (–15 to 46)	2 (–44 to 33)	37 (14 to 55)
**Outpatients**						
Cases, n (% vaccinated)	50 (14)	112 (26)	66 (14)	93 (38)	114 (32)	122 (24)
Influenza A(H1N1)pdm09	47	0	16	31	3	97
Influenza A(H3N2)	0	102	3	60	49	12
Influenza B	3	10	47	0	62	11
Controls, n (% vaccinated)	55 (31)	62 (32)	52 (33)	53 (32)	67 (25)	84 (39)
Crude VE, % (95% CI)	64 (3 to 86)	27 (–45 to 63)	67 (19 to 87)	–26 (–156 to 38)	–36 (–167 to 31)	52 (12 to 74)
Adjusted VE, % (95% CI) ^a^	84 (34 to 95)	58 (–20 to 85)	78 (4 to 89)	–35 (–231 to 45)	–66 (–280 to 27)	45 (–14 to 74)
**Inpatients**						
Cases, n (% vaccinated)	50 (40)	17 (47)	24 (33)	186 (49)	165 (58)	243 (46)
Influenza A(H1N1)pdm09	48	0	6	64	3	182
Influenza A(H3N2)	0	15	3	117	88	22
Influenza B	2	2	15	0	71	35
Controls, n (% vaccinated)	182 (58)	54 (52)	79 (56)	261 (61)	246 (60)	446 (61)
Crude VE, % (95% CI)	52 (9 to 75)	17 (–146 to 72)	60 (–4 to 85)	37 (8 to 57)	10 (–34 to 40)	45 (25 to 60)
Adjusted VE, % (95% CI) ^a^	36 (–32 to 68)	–4 (–485 to 82)	81 (28 to 95)	32 (–5 to 56)	16 (–31 to 46)	36 (8 to 56)

CI: confidence interval; VE: vaccine effectiveness.

^a^ Vaccine effectiveness adjusted by age groups (50–59, 60–69, 70–79 and ≥ 80 years), sex, major chronic conditions, functional dependence, hospitalisation in the previous 12 months, healthcare setting (primary healthcare and hospital), and month of sample collection.

### Average effectiveness of the influenza vaccine in six seasons

In the analysis of the six influenza seasons, the laboratory-confirmed cases had received the influenza vaccine in a lower proportion (39%) than the test–negative controls (53%; p < 0.001). In the adjusted analysis, the average VE was 34% (95% CI: 6 to 54) in preventing outpatient cases and 32% (95% CI: 15 to 45) in preventing hospitalised cases (Table 3 and Figure).

**Table 3:** Influenza vaccine effectiveness in preventing laboratory-confirmed cases by healthcare setting, age and comorbidity in northern Spain, 2010/11 to 2015/16

	All patients	Outpatients	Inpatients
**All swabbed patients**			
Cases, n (% vaccinated)	1,242 (39)	557 (26)	685 (49)
Controls, n (% vaccinated)	1,641 (53)	373 (32)	1,268 (60)
Crude VE, % (95% CI)	45 (36 to 53)	23 (2 to 45)	35 (22 to 46)
Adjusted VE, % (95% CI) ^a^	31 (18 to 43)	34 (6 to 54)	32 (15 to 45)
**Aged 50–64 years old**			
Cases, n (% vaccinated)	572 (16)	384 (13)	188 (21)
Controls, n (% vaccinated)	523 (27)	242 (20)	281 (32)
Crude VE, % (95% CI)	49 (31 to 62)	41 (9 to 62)	44 (14 to 34)
Adjusted VE, % (95% CI) ^a^	49 (29 to 64)	56 (29 to 73)	43 (5 to 66)
**Aged ≥ 65 years old**			
Cases, n (% vaccinated)	670 (58)	173 (55)	497 (59)
Controls, n (% vaccinated)	1,118 (66)	131 (55)	987 (67)
Crude VE, % (95% CI)	28 (12 to 41)	1 (–55 to 37)	29 (11 to 43)
Adjusted VE, % (95% CI) ^a^	25 (6 to 40)	3 (–71 to 45)	29 (9 to 44)
**Patients without major chronic conditions**			
Cases, n (% vaccinated)	459 (22)	336 (20)	123 (28)
Controls, n (% vaccinated)	414 (29)	203 (20)	211 (37)
Crude VE, % (95% CI)	31 (6 to 49)	3 (–49 to 37)	35 (–6 to 60)
Adjusted VE, % (95% CI) ^a^	32 (–1 to 54)	23 (–31 to 55)	42 (–15 to 71)
**Patients with major chronic conditions**			
Cases, n (% vaccinated)	783 (48)	221 (36)	562 (53)
Controls, n (% vaccinated)	1,227 (62)	170 (47)	1,057 (64)
Crude VE, % (95% CI)	42 (30 to 51)	38 (7 to 59)	36 (21 to 48)
Adjusted VE, % (95% CI) ^a^	32 (16 to 45)	41 (2 to 65)	30 (12 to 45)

CI: confidence interval; VE: vaccine effectiveness.

^a^ Vaccine effectiveness adjusted by age groups (50–59, 60–69, 70–79 and ≥ 80 years), sex, major chronic conditions, functional dependence, hospitalisation in the previous 12 months, healthcare setting (primary healthcare and hospital), and influenza season and month of sample collection.

**Figure:**
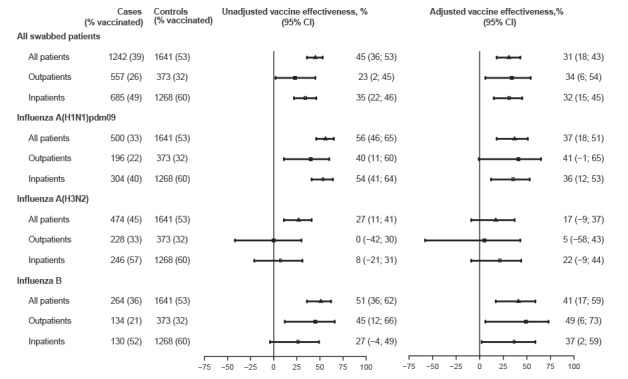
Influenza vaccine effectiveness in preventing laboratory-confirmed cases by (sub)type of virus and healthcare setting in northern Spain, 2010/11 to 2015/16

CI: confidence interval.

Vaccine effectiveness adjusted by age groups (50–59, 60–69, 70–79 and ≥ 80 years), sex, major chronic conditions, functional dependence, hospitalisation in the previous 12 months, healthcare setting (primary healthcare and hospital), and influenza season and month of sample collection.

VE in preventing laboratory-confirmed influenza A(H1N1)pdm09 was 41% (95% CI: –1 to 65) for outpatient cases and 36% (95% CI: 12 to 53) for hospitalised cases, and was 49% (95% CI: 6 to 73) and 37% (95% CI: 2 to 59), respectively, to prevent influenza B. Nevertheless, a low and non-significant effect was observed for the prevention of outpatient and inpatient cases of influenza A(H3N2) (Figure).

The adjusted VE estimates did not show relevant differences by pre-existing comorbidity. Although the interaction terms did not reach statistical significance, the adjusted VE estimate seemed to be lower in outpatients aged 65 years or older than in those aged 50–64 years (3% vs 56%; p = 0.064), but this difference nearly disappeared in hospitalised cases (29% vs 43%; p = 0.524) (Table 3).

In the analysis of the general practice setting, adults aged 65 years or older had lower adjusted VE than those aged 50–64 years against influenza A(H3N2) (–48% vs 40%; p = 0.155) and influenza A(H1N1)pdm09 (–20% vs 63%; p = 0.068), although both comparisons did not reach statistical significance. A similar difference was not observed in influenza B (62% vs 55%; p = 0.892). In the analysis of hospitalised patients such declines in VE with age were not observed (Table 4).

**Table 4:** Influenza vaccine effectiveness in preventing laboratory-confirmed cases by healthcare setting, age group and virus (sub)type, northern Spain, 2010/11 to 2015/16

	Age 50–64 years			Age ≥ 65 years		
	All patients	Outpatients	Inpatients	All patients	Outpatients	Inpatients
**Influenza A(H1N1)pdm09**						
Cases, n (% vaccinated)	273 (15)	148 (12)	125 (19)	227 (55)	48 (56)	179 (55)
Controls, n (% vaccinated)	523 (27)	242 (20)	281 (32)	1,118 (66)	131 (55)	987 (67)
Crude VE, % (95% CI)	51 (28 to 67)	49 (7 to 72)	50 (16 to 70)	36 (15 to 52)	–1 (–95 to 48)	40 (18 to 57)
Adjusted VE, % (95% CI) ^a^	56 (31 to 72)	63 (26 to 82)	47 (2 to 72)	25 (–6 to 46)	–20 (–200 to 52)	31 (1 to 52)
**Influenza A(H3N2)**						
Cases, n (% vaccinated)	175 (17)	138 (15)	37 (24)	299 (62)	90 (59)	209 (63)
Controls, n (% vaccinated)	523 (27)	242 (20)	281 (32)	1118 (66)	131 (55)	987 (67)
Crude VE, % (95% CI)	43 (11 to 63)	31 (–24 to 60)	32 (–51 to 69)	16 (–10 to 35)	–17 (–102 to 32)	17 (–14 to 39)
Adjusted VE, % (95% CI) ^a^	36 (–12 to 63)	40 (–23 to 70)	39 (–71 to 78)	14 (–19 to 38)	–48 (–249 to 38)	21 (–12 to 45)
**Influenza B**						
Cases, n (% vaccinated)	124 (15)	98 (12)	26 (23)	140 (55)	36 (44)	104 (59)
Controls, n (% vaccinated)	523 (27)	242 (20)	281 (32)	1118 (66)	131 (55)	987 (67)
Crude VE, % (95% CI)	53 (20 to 73)	45 (–9 to 72)	36 (–64 to 75)	37 (9 to 55)	34 (–38 to 69)	31 (–4 to 54)
Adjusted VE, % (95% CI) ^a^	47 (–2 to 73)	55 (–9 to 81)	13 (–184 to 73)	41 (10 to 61)	62 (–7 to 87)	40 (3 to 63)

CI: confidence interval; VE: vaccine effectiveness.

^a^ Vaccine effectiveness adjusted by age groups 50–59, 60–69, 70–79 and ≥ 80 years), sex, major chronic conditions, functional dependence, hospitalisation in the previous 12 months, healthcare setting (primary healthcare and hospital), and influenza season and month of sample collection.

### Comparison of vaccine effectiveness between outpatient and inpatient cases

In the analysis of all influenza cases, a non-statistically significant decrease in the risk of hospitalisation among vaccinated cases was observed (adjusted OR 0.83; 95% CI: 0.59 to 1.18). Influenza vaccination was associated with a 54% reduction in hospitalisation among influenza A(H3N2) cases (adjusted OR 0.46; 95% CI: 0.24 to 0.90). Although other results did not reach statistical significance, influenza vaccination seemed to be associated with a decline in the risk of hospitalisation among influenza A(H1N1)pdm09 cases (adjusted OR 0.73; 95% CI: 0.42 to 1.27) and with an increase in hospital admissions among influenza B cases (adjusted OR 1.64; 95% CI: 0.75 to 3.59). The analyses stratified in two age groups were consistent for influenza A(H3N2); and, although non-statistically significant, the point estimates suggest a decline in the risk of hospitalisation of influenza A(H1N1)pdm09 cases aged 65 years or older. In the 2013/14 season dominated by influenza A(H3N2) virus, influenza vaccination was also associated with lower risk of hospitalisation among influenza cases (adjusted OR 0.49; 95% CI: 0.24 to 0.99) (Table 5).

**Table 5:** Comparison of the influenza vaccination status of inpatient vs outpatient influenza cases by influenza (sub)type, age group and season, northern Spain, 2010/11 to 2015/16

	Inpatient cases		Outpatient cases		AOR (95% CI)^a^	p value
**All ages and seasons**	**n**	**% vaccinated**	**n**	**% vaccinated**		
Total	685	49	557	26	0.83 (0.59 to 1.18)	0.304
Influenza A(H1N1)pdm09	304	40	196	22	0.73 (0.42 to 1.27)	0.268
Influenza A(H3N2)	246	57	228	33	0.46 (0.24 to 0.90)	0.023
Influenza B	130	52	134	21	1.64 (0.75 to 3.59)	0.215
**Age 50–64 years**						
Total	188	21	384	13	1.03 (0.59 to 1.80)	0.919
Influenza A(H1N1)pdm09	125	19	148	12	1.17 (0.53 to 2.58)	0.698
Influenza A(H3N2)	37	24	138	15	0.32 (0.09 to 1.23)	0.099
Influenza B	26	23	98	12	2.36 (0.59 to 9.45)	0.227
**Age ≥ 65 years**						
Total	497	59	173	55	0.71 (0.44 to 1.14)	0.156
Influenza A(H1N1)pdm09	179	55	48	56	0.41 (0.16 to 1.03)	0.056
Influenza A(H3N2)	209	63	90	59	0.50 (0.22 to 1.12)	0.092
Influenza B	104	59	36	44	1.81 (0.63 to 5.17)	0.269
**Influenza season**						
2010/11	50	40	50	14	3.03 (0.83 to 11.12)	0.095
2011/12	17	47	112	26	0.40 (0.09 to 1.88)	0.245
2012/13	24	33	66	14	1.17 (0.15 to 8.95)	0.877
2013/14	186	49	93	38	0.49 (0.24 to 0.99)	0.048
2014/15	165	58	114	32	0.91 (0.45 to 1.83)	0.797
2015/16	243	46	122	24	0.87 (0.45 to 1.67)	0.668

AOR: adjusted odds ratio; CI: confidence interval.

^a^ OR and 95% CI adjusted by age groups (50–59, 60–69, 70–79 and ≥ 80 years), sex, major chronic conditions, functional dependence, hospitalisation in the previous 12 months, and influenza season and month of sample collection.

The comparison of hospitalised controls vs outpatient controls did not find an association with vaccine status in the adjusted analysis (adjusted OR 1.01; 95% CI: 0.72 to 1.41), thus indicating successful control of potential confounding.

## Discussion

During six influenza seasons from 2010/11 to 2015/16, the trivalent inactivated influenza VE was on average moderate and similar in preventing laboratory-confirmed influenza in general practice (34%) and hospital settings (32%), with both outcomes evaluated at the same time and in the same population of older adults. VE was moderate against cases of influenza A(H1N1)pdm09 (37%) and influenza B (41%), and was low against cases infected with Influenza A(H3N2) virus (17%). These results are consistent with the results of a recent systematic review [28].

In the majority of situations evaluated in our study, the same type and brand of influenza vaccine in the same season was equally effective in preventing laboratory-confirmed influenza cases that required outpatient assistance and those requiring hospitalisation. These results confirm those of a Spanish multicentre study that compared VE in outpatient and inpatient settings in the same areas in a single influenza season [8], as well as the analysis of 25 pairs of VE estimates from parallel studies in inpatient and outpatient settings in the same influenza season, in the same country, and in similar age groups, although only three of these pairs covered both settings in the same population [10]. Our results are also consistent with the absence of association between influenza vaccination and hospital admissions within 14 days after illness onset reported among outpatient cases with laboratory-confirmed influenza [29]. All this evidence argues against the hypothesis that, in general, influenza vaccination mitigates influenza illness severity and reduces the risk of hospital admission in people in whom it did not prevent influenza infection.

Nevertheless, in some analyses we observed a different behaviour. The vaccine was not effective in preventing outpatient cases of influenza A(H3N2), but did provide some protection (22%) against hospitalisations. This difference in VE was demonstrated in the case-to-case comparison and was also observed in the specific analysis of the 2013/14 season when A(H3N2) virus predominated and VE was very low. A higher protection of the vaccine against hospitalisations was also suggested in the analysis of influenza A(H1N1)pdm09 cases aged 65 years or older.

Recently, Petrie et al. [30] reported higher VE in preventing hospitalisations than that reported from similar studies in ambulatory care settings, in a season with circulating influenza A(H3N2) viruses that were antigenically drifted from the vaccine virus. In our study the higher VE among hospitalised influenza cases was associated with very low or no VE, type A influenza virus, and older patients. In situations of low vaccine-induced immunity against the circulating viruses, the relative importance of the vaccine effect in mitigating the severity of illness in vaccine failures would increase. This additional benefit of the vaccine cannot be detected in studies based on general practice settings only.

The average effect of the vaccine over all six seasons seemed to be lower in adults aged 65 or older than in those aged 50–64 years, which could be explained by immunosenescence [11]. Interestingly, this reduced VE with increasing age was pronounced in outpatient cases with influenza type A, but was hardly seen in inpatient cases or in cases of influenza B. This finding has a positive component since this loss of VE with age would be smaller in more severe forms of influenza. All these results suggest that, even though on average influenza VE is similar in both healthcare settings, in some situations involving influenza A virus infections, elderly patients and low VE, there may be important differences in VE between patients in outpatient and inpatient settings, that can also be interpreted as an additional vaccine effect that reduces the risk of hospital admission in people in whom the vaccine fails to prevent influenza infection.

The joint analysis of primary healthcare patients and hospitalised patients recruited in the same season and region shows the effect of the vaccine on a more complete clinical spectrum of the disease, including mild, moderate and severe cases, and on a more varied patient profile. The absence of differences in vaccination status in the adjusted analysis of outpatient and inpatient controls reduces the difficulties in interpreting joint estimations of VE.

This study has several strengths. We used the test–negative design nested in a population-based cohort in both the general practice and hospital settings, which allows evaluation and comparison of VE against outpatient and inpatient influenza cases. All cases were laboratory-confirmed, and the controls tested negative for influenza. The outpatient and hospitalised patients were recruited in the same population and in the same influenza seasons, received the same type and brand of vaccine, and were exposed to the same circulating viruses. To compare VE in both settings we directly compared the vaccination status of inpatient cases vs outpatient cases, which eliminates possible problems of comparability between the two control groups and, interestingly, reveals the vaccine effect in preventing hospitalisations among laboratory-confirmed influenza cases [8]. The analysis of six seasons increased the power of the study and achieved sufficient representation of different virus (sub)types and patient characteristics. The variability due to vaccine type was minimal since only one product was used in the vaccination programme in each season.

This study had some possible limitations. Although restriction to adults above 50 years of age considerably improved the comparability between outpatient and inpatient cases, there nevertheless remained differences that could hamper sufficient control of confounding factors. Moreover, we cannot totally discount the ‘healthy vaccinee effect’ whereby frail patients may be less likely to be vaccinated and more likely to be admitted to hospital [31]. However, this does not seem to have occurred since the adjusted model that compared inpatient controls with outpatient controls did not find an association between vaccination status and healthcare setting. We included adults aged 50–59 years, in whom vaccination is recommended and offered free of charge only if they have a chronic condition; however, all analyses were adjusted or stratified by age, and this did not seem to have an important effect on the results.

Estimates in hospitalised patients may be biased if they are diagnosed with a longer delay from the time of infection; however, in our study this bias was reduced as access to hospital emergency rooms was unrestricted, admission was based only on medical judgment, and there was a protocol for early swabbing and testing of ILI cases before admission.

Hospitalised cases were older and had more comorbidities than outpatient cases, and this may increase the risk of false negative results due to reduced viral shedding with age [32]. We reduced this bias by double swabbing of patients, restricting the analysis to older adults, and adjusting by age groups in the case-to-case comparison.

Although we analysed six influenza seasons and the number of patients included was high, the statistical power in some analyses (by season or age subgroup) was low; therefore caution should be exercised when explaining some results.

The effect of prior vaccination has arisen as a relevant factor in understanding influenza VE, but addressing this issue requires specifically focused studies [22,33].

In conclusion, influenza vaccination was on average moderately effective in preventing laboratory-confirmed influenza over six seasons in northern Spain. In general, VE was similar in preventing outpatient cases and hospitalisations with influenza, although in some situations involving influenza A virus infection, elderly patients and low vaccine effectiveness, influenza vaccination may have an additional effect in reducing the risk of hospital admission in people in whom the vaccine fails to prevent influenza infection. This effect increases the total benefit of the influenza vaccine and reinforces the recommendation of vaccination. The general practice and hospital settings provide complementary points of view for understanding the complete effect of the influenza vaccination. More studies linking both points of view are needed.

## Notes

**Members of the Primary Health Care Sentinel Network of Navarre:** I Abad, P Aldaz, E Álvarez, N Alvarez, JJ Arana, I Arceiz, E Arina, I Arribas, MD Artajo, B Azagra, FC Bartolome, C Bolea, A Brugos, B Cano, MV Castresana, JC Cenoz, F Cia, B Compains, F Cortés, B Churío, PC Cuevas, EM Da Costa, J Díez Espino, M Doiz, FJ Escribano, MJ Esparza, V Etayo, C Fernández Alfaro, B Flamarique, J Gamboa, ML Garcés, L García Blanco, AB German, A Giner, N Goñi, MJ Guillorme, JO Guiu, JC Gurbindo, MJ Guruchaga, JA Heras, MC Hijos, J Huidobro, S Indurain, B Iñigo, MC Irigoyen, JJ Jurio, MP León, JJ Longás, MJ López, MT Maquirriain, JJ Miner, M Moreno, MA Moros, U Navarro, FJ Orozco, M Orte, P Palacio, J Palau, C Pérez Lecumberri, P Pérez Pascual, B Pérez Sanz, A Prado, M Prado, A Puig, E Ridruejo, M Ramos, BE Rípodas, M Rodríguez, MA Roncal, I Ruiz Puertas, C Sánchez, P Sarrasqueta, MA Senosiain, J Sola, M Sota, ME Ursua, IA Urtasun, MJ Vigata, MT Virto, F Elía, E Albeniz.

**Members of the Network for Influenza Surveillance in Hospitals of Navarre:** P Artajo, X Beristain, J Chamorro, C Ezpeleta, P Fanlo, F Gil, J Hueto, C Martín, A Navascués, L Peña, I Polo, M Ruiz, N Viguria (Complejo Hospitalario de Navarra), M Fernández-Alonso, G Reina (Clínica Universidad de Navarra), JJ García Irure, M Ortega, M Torres (Hospital Reina Sofía, Tudela), F Lameiro, AI Álvaro (Hospital García Orcoyen, Estella), I Casado, A Zabala, M García Cenoz, J Díaz-González, L Fernandino, M Arriazu, A Barricarte, J Castilla (Instituto de Salud Pública de Navarra).

## References

[1] Glezen WP Serious morbidity and mortality associated with influenza epidemics. Epidemiol Rev. 1982;4(1):25-44. 10.1093/oxfordjournals.epirev.a036250 6754408

[2] Thompson WW Shay DK Weintraub E Brammer L Bridges CB Cox NJ Influenza-associated hospitalizations in the United States. JAMA. 2004;292(11):1333-40. 10.1001/jama.292.11.1333 15367555

[3] Reed C Chaves SS Daily Kirley P Emerson R Aragon D Hancock EB Estimating influenza disease burden from population-based surveillance data in the United States. PLoS One. 2015;10(3):e0118369. 10.1371/journal.pone.0118369 25738736 PMC4349859

[4] Osterholm MT Kelley NS Sommer A Belongia EA Efficacy and effectiveness of influenza vaccines: a systematic review and meta-analysis. Lancet Infect Dis. 2012;12(1):36-44. 10.1016/S1473-3099(11)70295-X 22032844

[5] Kissling E Valenciano M Buchholz U Larrauri A Cohen JM Nunes B Influenza vaccine effectiveness estimates in Europe in a season with three influenza type/subtypes circulating: the I-MOVE multicentre case-control study, influenza season 2012/13. Euro Surveill. 2014;19(6):20701. 10.2807/1560-7917.ES2014.19.6.20701 24556348

[6] Puig-Barberà J García-de-Lomas J Díez-Domingo J Arnedo-Pena A Ruiz-García M Limón-Ramírez R Influenza vaccine effectiveness in preventing influenza A(H3N2)-related hospitalizations in adults targeted for vaccination by type of vaccine: a hospital-based test-negative study, 2011-2012 A(H3N2) predominant influenza season, Valencia, Spain. PLoS One. 2014;9(11):e112294. 10.1371/journal.pone.0112294 25392931 PMC4230985

[7] Havers F Sokolow L Shay DK Farley MM Monroe M Meek J Case-control study of vaccine effectiveness in preventing laboratory-confirmed influenza hospitalizations in older adults, United States, 2010-11. Clin Infect Dis. 2016;63(10):1304-11. 10.1093/cid/ciw512 27486114

[8] Castilla J Godoy P Domínguez A Martínez-Baz I Astray J Martín V Influenza vaccine effectiveness in preventing outpatient, inpatient, and severe cases of laboratory-confirmed influenza. Clin Infect Dis. 2013;57(2):167-75. 10.1093/cid/cit194 23532475

[9] Darvishian M Bijlsma MJ Hak E van den Heuvel ER Effectiveness of seasonal influenza vaccine in community-dwelling elderly people: a meta-analysis of test-negative design case-control studies. Lancet Infect Dis. 2014;14(12):1228-39. 10.1016/S1473-3099(14)70960-0 25455990

[10] Feng S Cowling BJ Sullivan SG Influenza vaccine effectiveness by test-negative design - Comparison of inpatient and outpatient settings. Vaccine. 2016;34(14):1672-9. 10.1016/j.vaccine.2016.02.039 26920469 PMC4826670

[11] Reber AJ Chirkova T Kim JH Cao W Biber R Shay DK Immunosenescence and challenges of vaccination against influenza in the aging population. Aging Dis. 2012;3(1):68-90. 22500272 PMC3320806

[12] Sullivan SG Feng S Cowling BJ Potential of the test-negative design for measuring influenza vaccine effectiveness: a systematic review. Expert Rev Vaccines. 2014;13(12):1571-91. 10.1586/14760584.2014.966695 25348015 PMC4277796

[13] McLean HQ Thompson MG Sundaram ME Kieke BA Gaglani M Murthy K Influenza vaccine effectiveness in the United States during 2012-2013: variable protection by age and virus type. J Infect Dis. 2015;211(10):1529-40. 10.1093/infdis/jiu647 25406334 PMC4407759

[14] Skowronski DM Janjua NZ De Serres G Sabaiduc S Eshaghi A Dickinson JA Low 2012-13 influenza vaccine effectiveness associated with mutation in the egg-adapted H3N2 vaccine strain not antigenic drift in circulating viruses. PLoS One. 2014;9(3):e92153. 10.1371/journal.pone.0092153 24667168 PMC3965421

[15] Simpson CR Lone NI Kavanagh K Ritchie LD Robertson C Sheikh A Trivalent inactivated seasonal influenza vaccine effectiveness for the prevention of laboratory-confirmed influenza in a Scottish population 2000 to 2009. Euro Surveill. 2015;20(8):21043. 10.2807/1560-7917.ES2015.20.8.21043 25742433

[16] Pebody R Warburton F Andrews N Ellis J von Wissmann B Robertson C Effectiveness of seasonal influenza vaccine in preventing laboratory-confirmed influenza in primary care in the United Kingdom: 2014/15 end of season results. Euro Surveill. 2015;20(36):30013. 10.2807/1560-7917.ES.2015.20.36.30013 26535911

[17] Rondy M Launay O Puig-Barberà J Gefenaite G Castilla J de Gaetano Donati K European hospital IVE network 2012/13 influenza vaccine effectiveness against hospitalised influenza A(H1N1)pdm09, A(H3N2) and B: estimates from a European network of hospitals. Euro Surveill. 2015;20(2):21011. 10.2807/1560-7917.ES2015.20.2.21011 25613779

[18] Cheng AC Kotsimbos T Kelly PM FluCAN Investigators Influenza vaccine effectiveness against hospitalisation with influenza in adults in Australia in 2014. Vaccine. 2015;33(51):7352-6. 10.1016/j.vaccine.2015.10.016 26529066

[19] Turner N Pierse N Bissielo A Huang Q Radke S Baker M SHIVERS investigation team Effectiveness of seasonal trivalent inactivated influenza vaccine in preventing influenza hospitalisations and primary care visits in Auckland, New Zealand, in 2013. Euro Surveill. 2014;19(34):20884. 10.2807/1560-7917.ES2014.19.34.20884 25188614 PMC4627593

[20] Kelly HA Lane C Cheng AC Influenza vaccine effectiveness in general practice and in hospital patients in Victoria, 2011-2013. Med J Aust. 2016;204(2):76.e1-4. 10.5694/mja15.01017 26821109

[21] Martínez-Baz I Navascués A Pozo F Chamorro J Albeniz E Casado I Influenza vaccine effectiveness in preventing inpatient and outpatient cases in a season dominated by vaccine-matched influenza B virus. Hum Vaccin Immunother. 2015;11(7):1626-33. 10.1080/21645515.2015.1038002 25996366 PMC4514388

[22] McLean HQ Thompson MG Sundaram ME Meece JK McClure DL Friedrich TC Impact of repeated vaccination on vaccine effectiveness against influenza A(H3N2) and B during 8 seasons. Clin Infect Dis. 2014;59(10):1375-85. 10.1093/cid/ciu680 25270645 PMC4207422

[23] Martínez-Baz I Martínez-Artola V Reina G Guevara M Cenoz MG Morán J Effectiveness of the trivalent influenza vaccine in Navarre, Spain, 2010-2011: a population-based test-negative case-control study. BMC Public Health. 2013;13(1):191. 10.1186/1471-2458-13-191 23496887 PMC3599901

[24] Castilla J Martínez-Baz I Martínez-Artola V Reina G Pozo F García Cenoz M Decline in influenza vaccine effectiveness with time after vaccination, Navarre, Spain, season 2011/12. Euro Surveill. 2013;18(5):20388. 10.2807/ese.18.05.20388-en 23399423

[25] Castilla J Navascués A Fernández-Alonso M Reina G Albéniz E Pozo F Effects of previous episodes of influenza and vaccination in preventing laboratory-confirmed influenza in Navarre, Spain, 2013/14 season. Euro Surveill. 2016;20(22):30243. 10.2807/1560-7917.ES.2016.21.22.30243 27277013

[26] Instituto de Salud Pública y Laboral de Navarra. Protocolo de vacunación antigripal 2015-2016. [Influenza vaccination protocol 2015-2016]. Boletín Informativo. 2015;85:1-4. Spanish. Available from: https://www.navarra.es/NR/rdonlyres/AECCD760-AB2A-4841-818A-FA53478FD6DC/326813/BOL8515.pdf

[27] European Commission. Commission Decision of 28 April 2008 amending Decision 2002/253/EC laying down case definitions for reporting communicable diseases to the Community network under Decision No 2119/98/EC of the European Parliament and of the Council. Luxembourg: Official Journal of the European Union. 18.6.2008:L 159/46. Available from: http://eur-lex.europa.eu/LexUriServ/LexUriServ.do?uri=OJ:L:2008:159:0046:0090:EN:PDF

[28] Belongia EA Simpson MD King JP Sundaram ME Kelley NS Osterholm MT Variable influenza vaccine effectiveness by subtype: a systematic review and meta-analysis of test-negative design studies. Lancet Infect Dis. 2016;16(8):942-51. 10.1016/S1473-3099(16)00129-8 27061888

[29] McLean HQ Meece JK Belongia EA Influenza vaccination and risk of hospitalization among adults with laboratory confirmed influenza illness. Vaccine. 2014;32(4):453-7. 10.1016/j.vaccine.2013.11.060 24291201

[30] Petrie JG Ohmit SE Cheng CK Martin ET Malosh RE Lauring AS Influenza vaccine effectiveness against antigenically drifted influenza higher than expected in hospitalized adults: 2014-2015. Clin Infect Dis. 2016;63(8):1017-25. 10.1093/cid/ciw432 27369320

[31] Jackson LA Jackson ML Nelson JC Neuzil KM Weiss NS Evidence of bias in estimates of influenza vaccine effectiveness in seniors. Int J Epidemiol. 2006;35(2):337-44. 10.1093/ije/dyi274 16368725

[32] Carrat F Vergu E Ferguson NM Lemaitre M Cauchemez S Leach S Time lines of infection and disease in human influenza: a review of volunteer challenge studies. Am J Epidemiol. 2008;167(7):775-85. 10.1093/aje/kwm375 18230677

[33] Martínez-Baz I Casado I Navascués A Díaz-González J Aguinaga A Barrado L Effect of repeated vaccination with the same vaccine component against 2009 pandemic influenza A(H1N1) virus. J Infect Dis. 2017;215(6):847-55. 10.1093/infdis/jix055 28453845

